# Supplementary material for: One-carbon metabolism nutrients impact the interplay between DNA methylation and gene expression in liver, enhancing protein synthesis in Atlantic salmon
Source: Epigenetics. 2024 Feb 25;19(1):2318517. doi: 10.1080/15592294.2024.2318517 (PMC10900267; doi:10.1080/15592294.2024.2318517)
Supplement: supp.docx [file KEPI_A_2318517_SM6809.docx]

**One-carbon metabolism nutrients impact the interplay between DNA methylation and gene expression in liver, enhancing protein synthesis in Atlantic Salmon**

Takaya Saito, Marit Espe, Vibeke Vikeså, Christoph Bock, Tårn H. Thomsen, Anne‐Catrin Adam, Jorge M.O. Fernandes, and Kaja H. Skjaerven

**Supplementary tables**

**Table S1.** Composition of the experimental diets (g/kg).

| **Ctrl** | **1C+** |  | **1C+** |
| --- | --- | --- | --- |
| **Wheat** | 54.22 | 50.64 | 47.05 |
| **Wheat gluten** | 132.03 | 132.03 | 132.03 |
| **Sunflower meal** | 10.0 | 10.0 | 10.0 |
| **Dehulled faba beans** | 30.0 | 30.0 | 30.0 |
| **Pea concentrate** | 150.0 | 150.0 | 150.0 |
| **Soy protein concentrate** | 240.0 | 240.0 | 240.0 |
| **Krill meal** | 20.0 | 20.0 | 20.0 |
| **Fish meal** | 120.0 | 120.0 | 120.0 |
| **Rapeseed oil** | 81.24 | 81.24 | 81.24 |
| **Fish oil** | 126.9 | 126.9 | 126.9 |
| **Water** | 11.43 | 11.84 | 12.26 |
| **DL-methionine** | **0.05** | **3.12** | **6.19** |
| **Choline** | 0.92 | 0.92 | 0.92 |
| **NRC mineral mix** | 2.0 | 2.0 | 2.0 |
| **NRC Vitamin mix** | 1.0 | 1.0 | 1.0 |
| **Vitamin B12** | **0.156** | **0.179** | **0.203** |
| **Folate** | **0.023** | **0.053** | **0.083** |
| **Vitamin B6** | **0.077** | **0.107** | **0.137** |
| **Taurine** | 2.8 | 2.8 | 2.8 |
| **Micronutrients** | 17.16 | 17.17 | 17.19 |


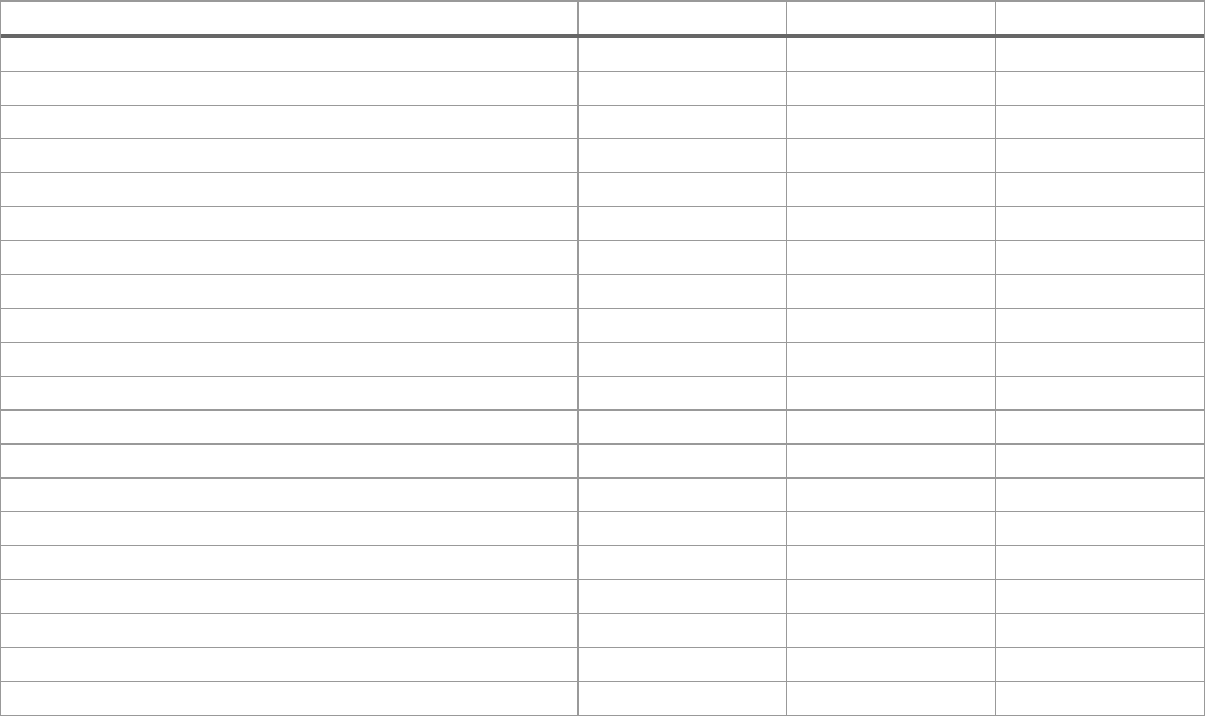


**Table S2.** Growth performance measured at four sampling points.

| **Sampling point** | **Measure^†^** | **Ctrl** |  | **1C+** |  | **1C++** |  | **p-value** |  |
| --- | --- | --- | --- | --- | --- | --- | --- | --- | --- |
| **S1** | BW (g) | 31.62±0.78 |  | 32.16±0.47 |  | 32.74±0.35 |  | 0.43 |  |
| **S2** | BW (g) | 85.81±3.14 |  | 90.54±1.24 |  | 87.59±4.64 |  | 0.62 |  |
|  | CF | 1.29±0.02 |  | 1.32±0.00 |  | 1.31±0.03 |  | 0.51 |  |
| **S3** | HSI | 1.09±0.04 |  | 0.99±0.00 |  | 1.02±0.01 |  | 0.06 |  |
|  | BW (g) | 90±3.67 |  | 93.73±2.42 |  | 97.70±4.23 |  | 0.36 |  |
| **S4** | BW (g) | 462.53±19.23 | **b** | 539.20±8.49 | **a** | 474.17±3.95 | **b** | **0.009** |  |
|  | CF | 1.46±0.01 | **b** | 1.55±0.01 | **a** | 1.53±0.02 | **a** | **0.006** |  |
|  | HSI | 1.64±0.08 | **b** | 1.32±0.04 | **a** | 1.42±0.05 | **ab** | **0.025** |  |


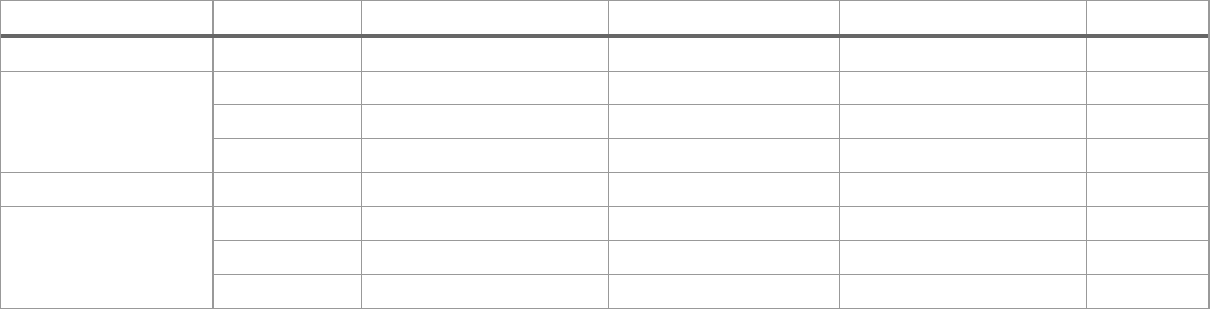


Mean values of three tanks are followed by SEM and the compact letter display of Tukey's post hoc test (p < 0.05, ANOVA followed by Tukey’s post hoc test).

^†^BW (body weight), CF (condition factor), and HSI (hepatosomatic index).

**Table S3.** Read counts of RNA-seq samples after initial quality control, alignment, and quantification.

| **Sample** | **Sex** | **Treatment** | **M Seqs^1^** | **M Aligned^2^** | **% Aligned^2^** | **M Assigned^3^** | **% Assigned^3^** |
| --- | --- | --- | --- | --- | --- | --- | --- |
| **1Cp1** | M | 1C+ | 14.6 | 12 | 82.1% | 10.8 | 58.9% |
| **1Cp2** | M | 1C+ | 11.8 | 9.6 | 81.8% | 8.6 | 57.5% |
| **1Cp3** | M | 1C+ | 16.8 | 13.7 | 81.7% | 12.3 | 58.2% |
| **1Cp4** | F | 1C+ | 14.4 | 11.7 | 80.7% | 10.5 | 55.4% |
| **1Cp5** | F | 1C+ | 15.8 | 12.9 | 81.8% | 11.7 | 58.5% |
| **1Cp6** | M | 1C+ | 14.5 | 11.6 | 79.8% | 10.4 | 53.9% |
| **1Cp7** | F | 1C+ | 16.8 | 13.7 | 81.9% | 12.3 | 58.6% |
| **1Cp8** | F | 1C+ | 16.8 | 13.2 | 78.5% | 12 | 56.4% |
| **1Cp9** | F | 1C+ | 15.5 | 12.7 | 82.1% | 11.4 | 58.8% |
| **1Cpp1** | F | 1C++ | 14.5 | 11.9 | 82.3% | 10.8 | 60.3% |
| **1Cpp2** | M | 1C++ | 16.5 | 13.4 | 81.2% | 12.2 | 57.7% |
| **1Cpp3** | F | 1C++ | 16.6 | 13.4 | 80.8% | 12.2 | 57.1% |
| **1Cpp4** | F | 1C++ | 16.1 | 13.2 | 82.1% | 11.8 | 57.8% |
| **1Cpp5** | F | 1C++ | 16.6 | 13.3 | 80.2% | 11.9 | 54.8% |
| **1Cpp6** | M | 1C++ | 14.3 | 11.6 | 81.3% | 10.4 | 56.9% |
| **1Cpp7** | F | 1C++ | 14.5 | 11.5 | 79.3% | 10.4 | 53.0% |
| **1Cpp8** | M | 1C++ | 14.6 | 12 | 82.2% | 10.8 | 58.9% |
| **1Cpp9** | M | 1C++ | 16.2 | 13.2 | 81.8% | 12 | 58.5% |
| **Ctrl1** | F | Ctrl | 17.1 | 13.9 | 81.4% | 12.5 | 57.3% |
| **Ctrl2** | F | Ctrl | 12.4 | 10.3 | 82.8% | 9.2 | 60.3% |
| **Ctrl3** | M | Ctrl | 15.4 | 12.6 | 81.8% | 11.3 | 58.1% |
| **Ctrl4** | F | Ctrl | 18 | 14.9 | 82.7% | 13.4 | 59.8% |
| **Ctrl5** | M | Ctrl | 15.8 | 13.1 | 82.9% | 11.8 | 60.4% |
| **Ctrl6** | M | Ctrl | 18.3 | 14.6 | 79.6% | 13 | 52.2% |
| **Ctrl7** | F | Ctrl | 15.5 | 12.9 | 83.1% | 11.6 | 60.4% |
| **Ctrl8** | M | Ctrl | 17.9 | 14.7 | 82.2% | 13.3 | 59.2% |
| **Ctrl9** | F | Ctrl | 16.4 | 13.3 | 81.3% | 11.9 | 56.5% |


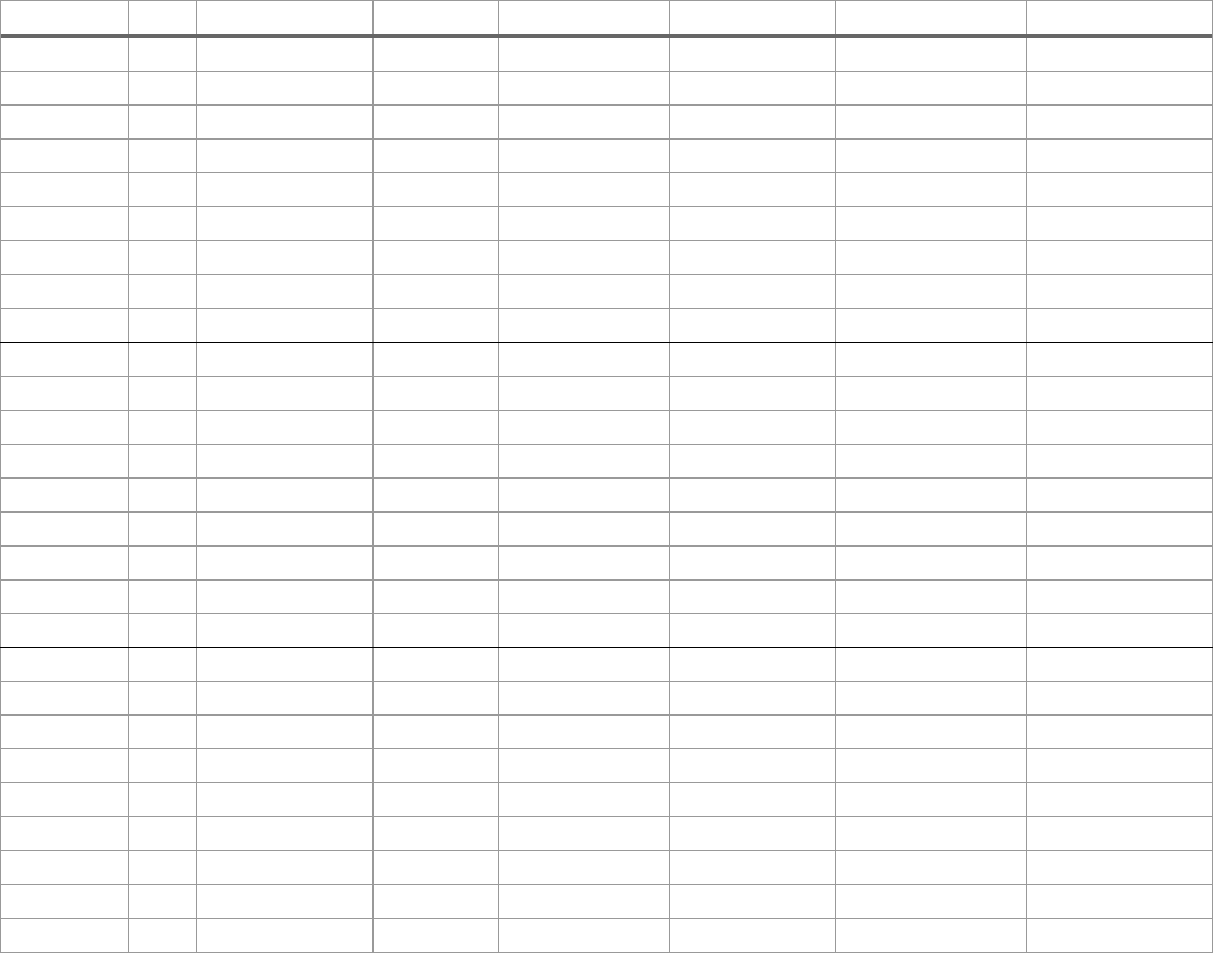


^1^Total read count after initial quality control by Trim Galore!.

^2^Count of aligned reads to the reference genome by STAR and the percentage of the aligned reads to the total reads.

^3^Count of the reads associated with known RNAs by featureCount with the percentage of the assigned reads among the total aligned sites, which include both unique and multiple aligned reads.

**Table S4.** Number of DEGs identified by three comparisons.

| **Comparison** | **Control** | **# DEGs** | **# Down-regulated** | **# Up-regulated** |
| --- | --- | --- | --- | --- |
| **1C+ vs Ctrl** | Ctrl | 874 | 513 | 361 |
| **1C++ vs Ctrl** | Ctrl | 759 | 395 | 364 |
| **1C++ vs 1C+** | 1C+ | 20 | 10 | 10 |


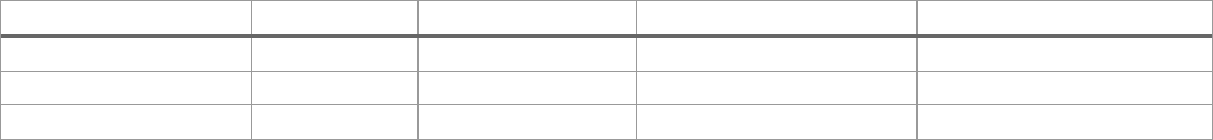


**Table S5.** Enriched KEGG pathways for the genes in DEG C1 and DEG C2 clusters by ORA.

| **Cluster** | **ID** | **Description** | **GeneRatio^1^** | **BgRatio^3^** | **p.adjust^3^** | **GSEA^4^** |  |
| --- | --- | --- | --- | --- | --- | --- | --- |
| **DEG C1** | sasa01240 | Biosynthesis of cofactors | 17/213 | 201/7462 | 7.48E-03 | Y |  |
|  | sasa00270 | Cysteine and methionine | 10/213 | 86/7462 | 1.03E-02 | Y |  |
|  |  | metabolism |  |  |  |  |  |
|  | sasa00220 | Arginine biosynthesis | 6/213 | 34/7462 | 1.54E-02 | N |  |
|  | sasa01230 | Biosynthesis of amino acids | 11/213 | 127/7462 | 2.98E-02 | Y |  |
|  | sasa00982 | Drug metabolism - cytochrome | 6/213 | 42/7462 | 2.98E-02 | N |  |
| **DEG C2** |  | P450 |  |  |  |  |  |
|  | sasa00591 | Linoleic acid metabolism | 4/144 | 19/7462 | 2.15E-02 | Y |  |
|  | sasa04141 | Protein processing in | 16/144 | 321/7462 | 2.15E-02 | Y |  |
|  |  | endoplasmic reticulum |  |  |  |  |  |
|  | sasa03060 | Protein export | 5/144 | 40/7462 | 3.15E-02 | Y |  |
|  | sasa00980 | Metabolism of xenobiotics by | 5/144 | 43/7462 | 3.30E-02 | N |  |
|  |  | cytochrome P450 |  |  |  |  |  |


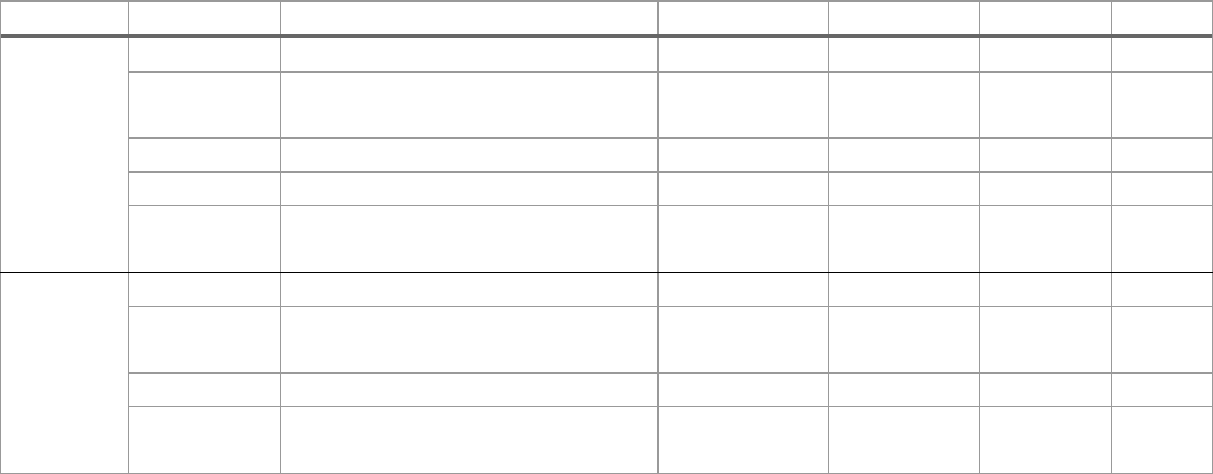


^1,2,3^Output of the enrichKEGG function provided by the clusterProfiler package. GeneRatio: gene ratio, BgRatio:

background ratio, p.adjust: adjusted p-value by the Benjamini-Hochberg procedure.

^4^Y: the pathway is also enriched by one of the GSEA results. N: the pathway is not enriched by GSEA.

**Table S6.** Enriched KEGG pathways for the C1+ vs Ctrl comparison by GSEA.

| **ID** | **Description** | **setSize^1^** | **NES^2^** | **p.adjus^3^** | **Support^4^** |  |
| --- | --- | --- | --- | --- | --- | --- |
| **sasa03010** | Ribosome | 255 | 2.09E+00 | 5.33E-09 | GSEA |  |
| **sasa04141** | Protein processing in endoplasmic reticulum | 378 | 1.95E+00 | 5.33E-09 | ORA, |  |
| **sasa04110** |  |  |  |  | GSEA |  |
|  | Cell cycle | 275 | -1.94E+00 | 5.33E-09 | GSEA |  |
| **sasa04115** | p53 signaling pathway | 145 | -1.82E+00 1.04E-04 | |  |  |
| **sasa04510** | Focal adhesion | 470 | 1.48E+00 | 1.51E-04 |  |  |
| **sasa03060** | Protein export | 44 | 2.10E+00 | 1.70E-04 | ORA |  |
| **sasa04216** | Ferroptosis | 108 | -1.84E+00 1.84E-04 | |  |  |
| **sasa04260** | Cardiac muscle contraction | 191 | 1.72E+00 | 1.84E-04 |  |  |
| **sasa04068** | FoxO signaling pathway | 331 | -1.58E+00 | 2.24E-04 |  |  |
| **sasa04218** | Cellular senescence | 373 | -1.57E+00 | 2.24E-04 |  |  |
| **sasa04914** | Progesterone-mediated oocyte maturation | 203 | -1.67E+00 2.58E-04 | |  |  |
| **sasa00190** | Oxidative phosphorylation | 245 | 1.61E+00 | 3.20E-04 |  |  |
| **sasa00100** | Steroid biosynthesis | 31 | -2.02E+00 | 5.46E-04 | GSEA |  |
| **sasa01230** | Biosynthesis of amino acids | 167 | -1.64E+00 | 7.34E-04 | ORA |  |
| **sasa00240** | Pyrimidine metabolism | 103 | -1.74E+00 | 7.68E-04 |  |  |


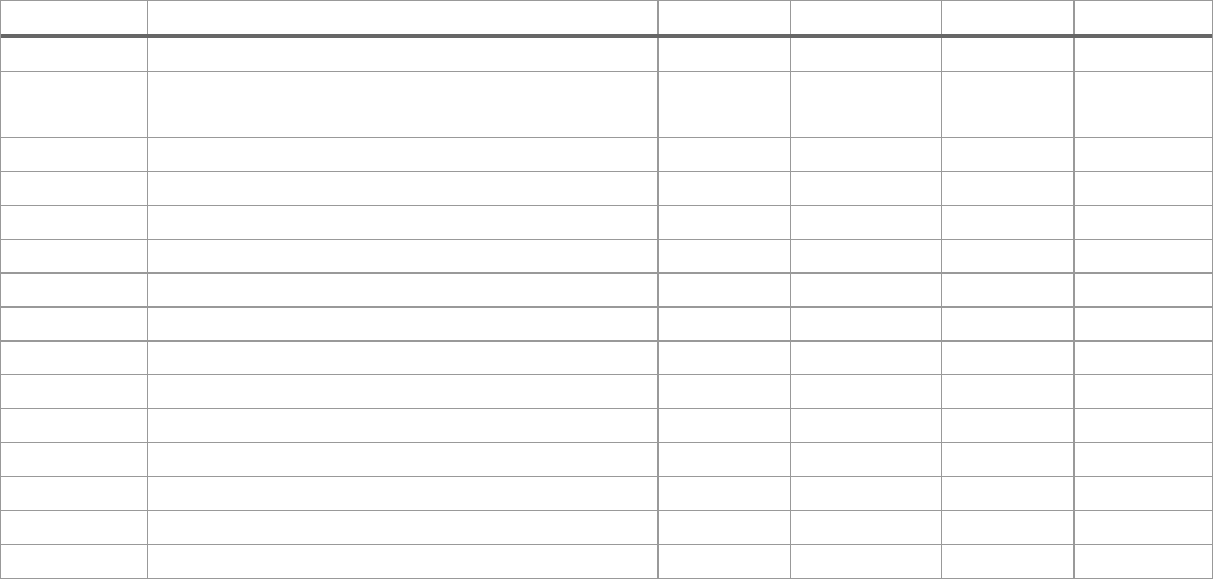


^1,2,3^Output of the gseKEGG function provided by the clusterProfiler package. setSize: the number of genes that

contributed for enrichment, NES: normalized enrichment score that indicates up-regulation (positive) or down-

regulation (negative), p.adjust: adjusted p-value by the Benjamini-Hochberg procedure.

^4^ORA: the pathway is also enriched by ORA. GSEA: the pathway is also enriched by at least one of the other GSEA results.

**Table S7.** Enriched KEGG pathways for the C1++ vs Ctrl comparison by GSEA.

| **ID** | **Description** | **setSize^1^** | **NES^2^** | **p.adjust^3^** | **Support^4^** |
| --- | --- | --- | --- | --- | --- |
| **sasa03010** | Ribosome | 255 | 2.63E+00 | 1.60E-08 | GSEA |
| **sasa00970** | Aminoacyl-tRNA biosynthesis | 62 | -2.26E+00 | 4.86E-07 | GSEA |
| **sasa04110** | Cell cycle | 275 | -1.83E+00 | 1.52E-06 | GSEA |
| **sasa00190** | Oxidative phosphorylation | 247 | 1.79E+00 | 6.07E-06 |  |
| **sasa03030** | DNA replication | 55 | -2.01E+00 | 1.47E-04 |  |
| **sasa01230** | Biosynthesis of amino acids | 164 | -1.81E+00 | 1.47E-04 |  |
| **sasa00270** | Cysteine and methionine metabolism | 107 | -1.85E+00 | 1.87E-04 |  |
| **sasa00100** | Steroid biosynthesis | 32 | -2.04E+00 | 3.83E-04 | GSEA |
| **sasa01232** | Nucleotide metabolism | 164 | -1.72E+00 | 5.59E-04 |  |
| **sasa01240** | Biosynthesis of cofactors | 260 | -1.62E+00 | 5.59E-04 | ORA |


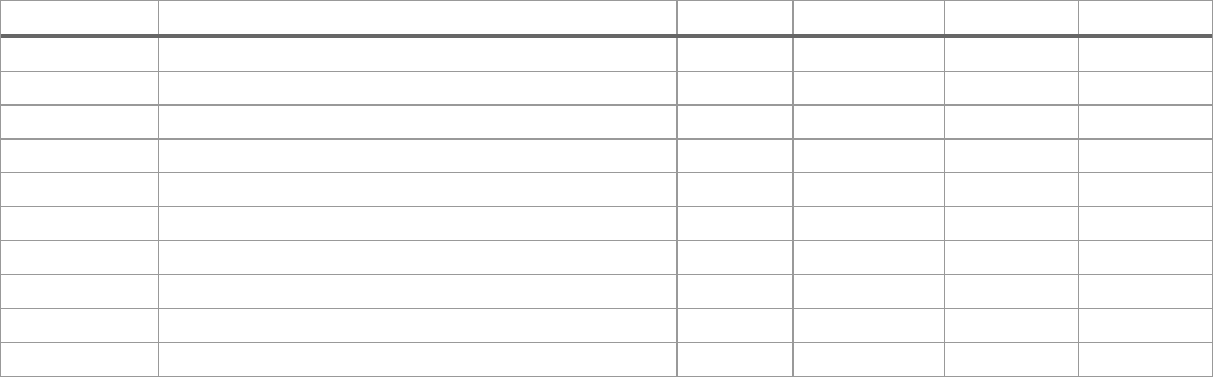


^1,2,3^Output of the gseKEGG function provided by the clusterProfiler package. setSize: the number of genes that

contributed for enrichment, NES: normalized enrichment score that indicates up-regulation (positive) or down-

regulation (negative), p.adjust: adjusted p-value by the Benjamini-Hochberg procedure.

^4^ORA: the pathway is also enriched by ORA. GSEA: the pathway is also enriched by at least one of the other GSEA results.

**Table S8.** Enriched KEGG pathways for the C1+ vs Ctrl comparison by GSEA.

| **ID** | **Description** | **setSize^1^** | **NES^2^** | **p.adjust^3^** | **Support^4^** |  |
| --- | --- | --- | --- | --- | --- | --- |
| **sasa03010** | Ribosome | 257 | 1.88E+00 | 3.43E-07 | GSEA |  |
| **sasa00970** | Aminoacyl-tRNA biosynthesis | 61 | -2.24E+00 | 4.56E-07 | GSEA |  |
| **sasa03015** | mRNA surveillance pathway | 179 | -1.66E+00 | 2.76E-04 |  |  |
| **sasa04141** | Protein processing in endoplasmic | 375 | -1.54E+00 | 4.17E-04 | ORA,GSEA |  |
| **sasa03013** | reticulum |  |  |  |  |  |
|  | Nucleocytoplasmic transport | 209 | -1.67E+00 | 4.33E-04 |  |  |


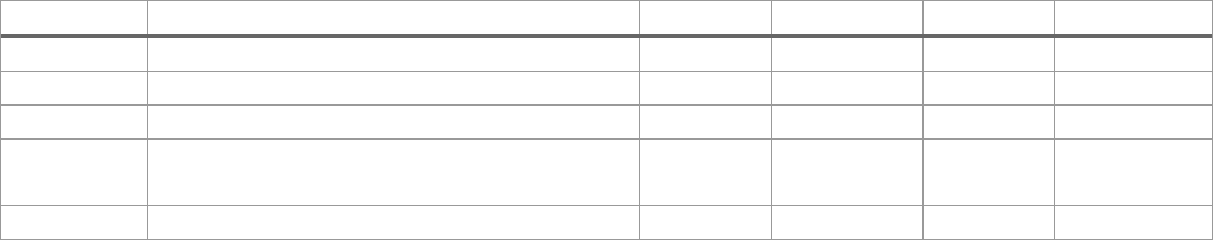


^1,2,3^Output of the gseKEGG function provided by the clusterProfiler package. setSize: the number of genes that

contributed for enrichment, NES: normalized enrichment score that indicates up-regulation (positive) or down-

regulation (negative), p.adjust: adjusted p-value by the Benjamini-Hochberg procedure.

^4^ORA: the pathway is also enriched by ORA. GSEA: the pathway is also enriched by at least one of the other GSEA results.

**Table S9.** Read counts of RRBS samples after initial quality control and alignment percentage.

| **Sample Name** | **Sex** | **Treatment** | **M Seqs^1^** | **% Aligned^2^** |
| --- | --- | --- | --- | --- |
| **1Cp1** | F | 1C+ | 67.4 | 47.5% |
| **1Cp2** | F | 1C+ | 44.7 | 47.5% |
| **1Cp3** | M | 1C+ | 74.2 | 47.1% |
| **1Cp4** | F | 1C+ | 46.7 | 47.1% |
| **1Cp5** | M | 1C+ | 51.6 | 47.8% |
| **1Cp6** | M | 1C+ | 49.1 | 47.1% |
| **1Cp7** | F | 1C+ | 42.3 | 46.7% |
| **1Cp8** | M | 1C+ | 66.3 | 47.0% |
| **1Cp9** | F | 1C+ | 58.6 | 46.8% |
| **1Cpp1** | M | 1C++ | 61.8 | 48.3% |
| **1Cpp2** | M | 1C++ | 61.6 | 49.0% |
| **1Cpp3** | M | 1C++ | 54.4 | 48.1% |
| **1Cpp4** | F | 1C++ | 66.3 | 47.5% |
| **1Cpp5** | F | 1C++ | 47.8 | 48.4% |
| **1Cpp6** | M | 1C++ | 36.5 | 47.7% |
| **1Cpp7** | F | 1C++ | 59.6 | 46.9% |
| **1Cpp8** | F | 1C++ | 50.7 | 46.4% |
| **1Cpp9** | F | 1C++ | 59.7 | 47.2% |
| **Ctrl1** | F | Ctrl | 63 | 48.0% |
| **Ctrl2** | M | Ctrl | 59 | 47.8% |
| **Ctrl3** | F | Ctrl | 65.9 | 47.9% |
| **Ctrl4** | F | Ctrl | 42.4 | 47.0% |
| **Ctrl5** | F | Ctrl | 60.3 | 46.7% |
| **Ctrl6** | M | Ctrl | 50.5 | 46.7% |
| **Ctrl7** | F | Ctrl | 46.4 | 47.4% |
| **Ctrl8** | M | Ctrl | 55 | 47.9% |
| **Ctrl9** | M | Ctrl | 45.4 | 47.6% |


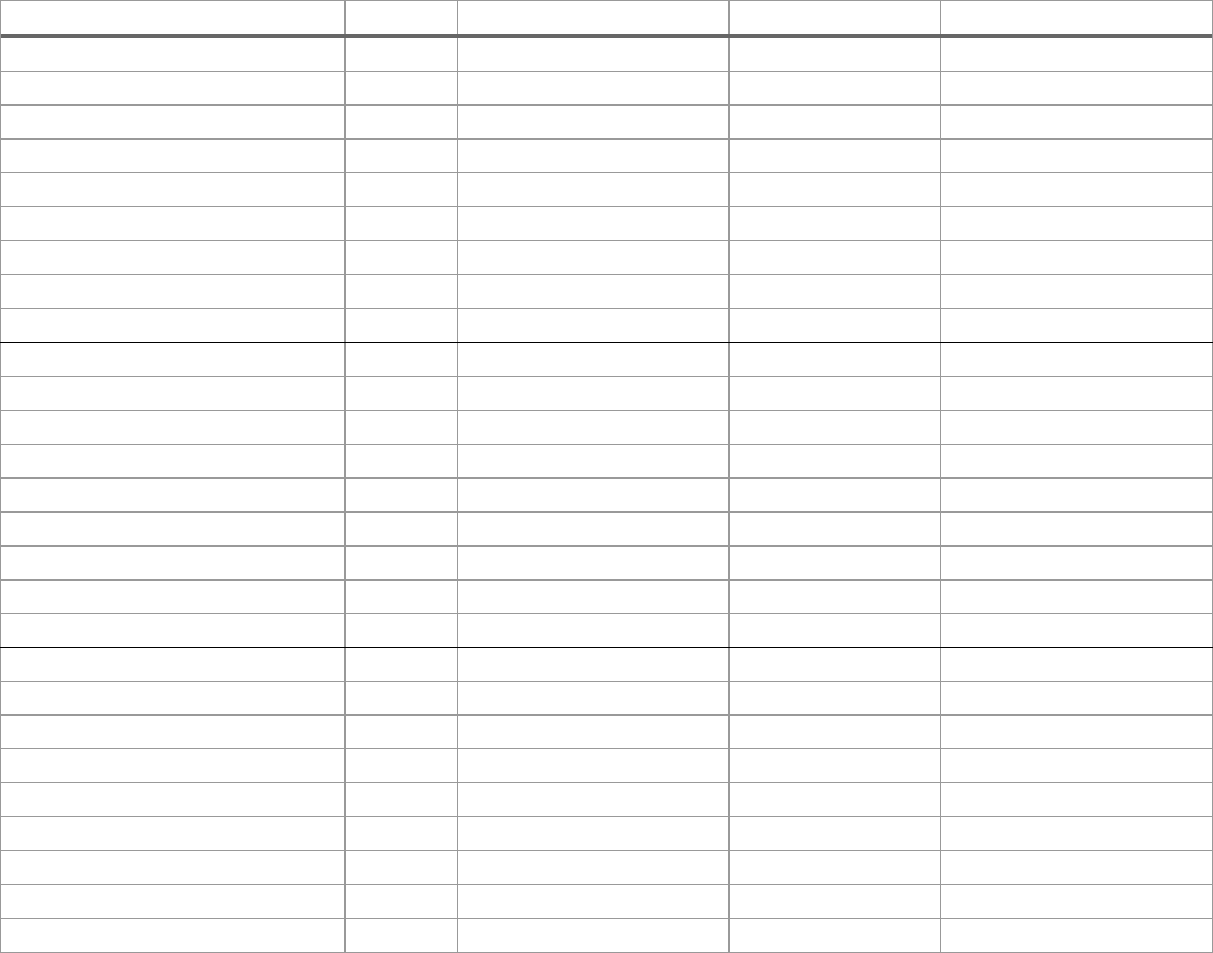


^1^Total read count after initial quality control by Trim Galore!.

- Percentage of the aligned reads to the reference genome by Bismark.

**Table S10.** Comparisons of two methylation rate distributions in different regions by KS tests.

| **Region** | **Size** | **X** | **Y** | **Alternative** | **p-value** | **Significance^†^** |
| --- | --- | --- | --- | --- | --- | --- |
| **All mapped CpGs** | 157 201 | 1C+ | Ctrl | **less** | **0** | ***** |
|  |  |  |  | greater | 0.81 |  |
|  |  | 1C++ | Ctrl | **less** | **0** | ***** |
|  |  |  |  | greater | 0.93 |  |
|  |  | 1C+ | 1C++ | **less** | **0** | ***** |
|  |  |  |  | greater | 0.85 |  |
| **GB** | 87 215 | 1C+ | Ctrl | **less** | **0** | ***** |
|  |  |  |  | greater | 0.83 |  |
|  |  | 1C++ | Ctrl | **less** | **0** | ***** |
|  |  |  |  | greater | 0.91 |  |
|  |  | 1C+ | 1C++ | **less** | **8.88e-16** | ***** |
|  |  |  |  | greater | 0.85 |  |
| **P** | 3 091 | 1C+ | Ctrl | **less** | **0.04** | ***** |
|  |  |  |  | greater | 0.81 |  |
|  |  | 1C++ | Ctrl | **less** | **0.02** | ***** |
|  |  |  |  | greater | 0.9 |  |
|  |  | 1C+ | 1C++ | less | 0.72 |  |
|  |  |  |  | greater | 0.47 |  |
| **Flank** | 48 148 | 1C+ | Ctrl | **less** | **0** | ***** |
|  |  |  |  | greater | 0.88 |  |
|  |  | 1C++ | Ctrl | **less** | **0** | ***** |
|  |  |  |  | greater | 0.84 |  |
|  |  | 1C+ | 1C++ | **less** | **9.55e-10** | ***** |
|  |  |  |  | greater | 0.93 |  |
| **IGR** | 47 748 | 1C+ | Ctrl | **less** | **0** | ***** |
|  |  |  |  | greater | 0.93 |  |
|  |  | 1C++ | Ctrl | **less** | **0** | ***** |
|  |  |  |  | greater | 0.98 |  |
|  |  | 1C+ | 1C++ | **less** | **0** | ***** |
|  |  |  |  | greater | 0.94 |  |


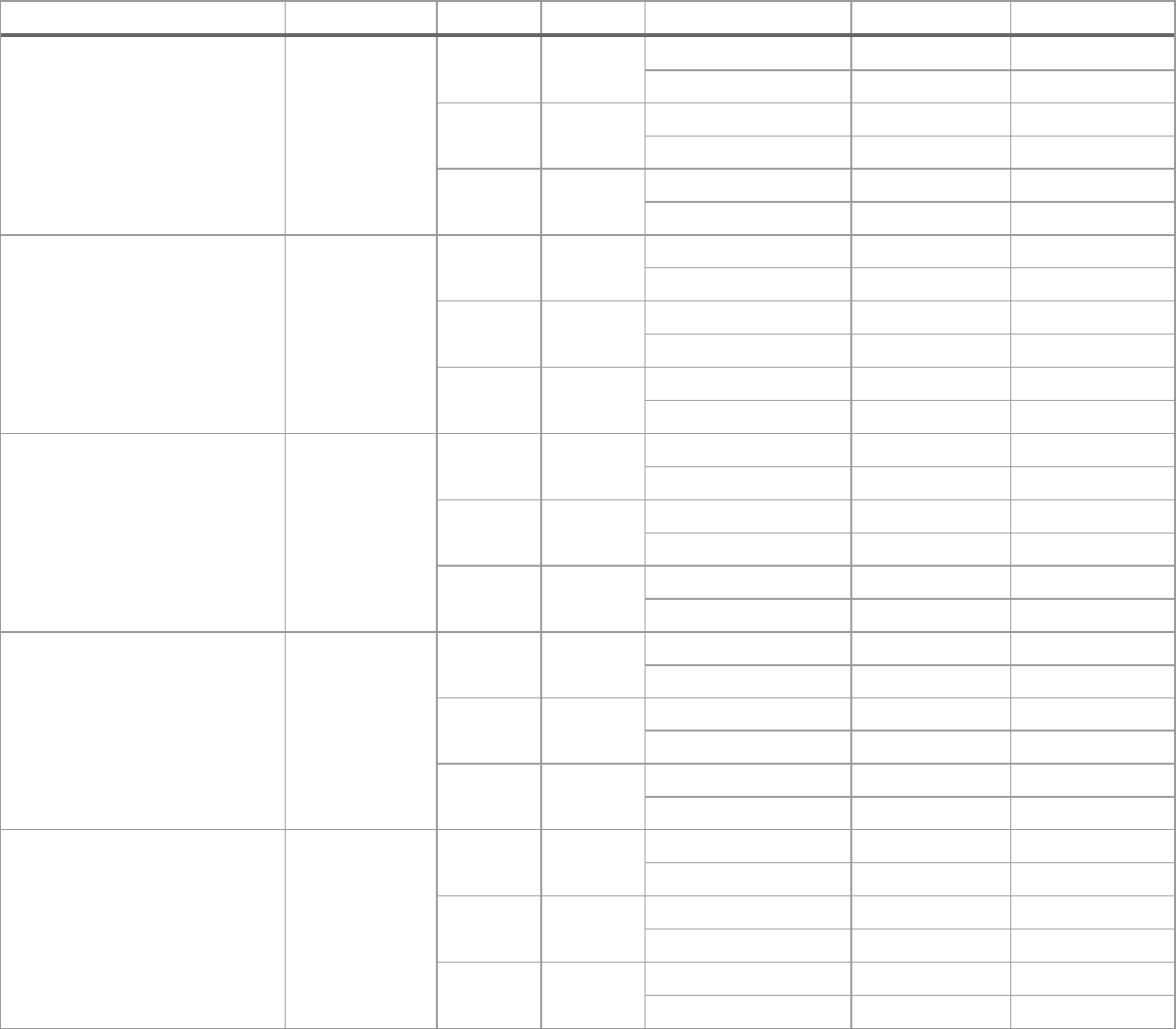


^†^’*’ indicates that KS test result is statistically significant with p-value < 0.05.

**Table S11.** Number of DMCs identified by three comparisons in four different regions.

| **Comparison** | **Region** | **#Mapped CpGs** | **#DMCs** | **(%)^1^** | **#DMCs (hypo)^2^** | **#DMCs (hyper)^3^** |  |
| --- | --- | --- | --- | --- | --- | --- | --- |
| **1C+ vs Ctrl** | GB | 107366 | 3061 | 2.9% | 933 | 2128 |  |
|  | P | 4189 | 154 | 3.7% | 49 | 105 |  |
|  | Flank | 74773 | 2390 | 3.2% | 793 | 1597 |  |
| **1C++ vs Ctrl** | IGR | 67078 | 2112 | 3.1% | 636 | 1476 |  |
|  | GB | 108055 | 2969 | 2.7% | 978 | 1991 |  |
|  | P | 4110 | 131 | 3.2% | 58 | 73 |  |
|  | Flank | 75504 | 2145 | 2.8% | 729 | 1416 |  |
| **1C++ vs 1C+** | IGR | 67018 | 2044 | 3% | 652 | 1392 |  |
|  | GB | 105087 | 2488 | 2.4% | 1359 | 1129 |  |
|  | P | 4103 | 134 | 3.3% | 84 | 50 |  |
|  | Flank | 74515 | 1973 | 2.6% | 1048 | 925 |  |
|  | IGR | 65076 | 1766 | 2.7% | 967 | 799 |  |


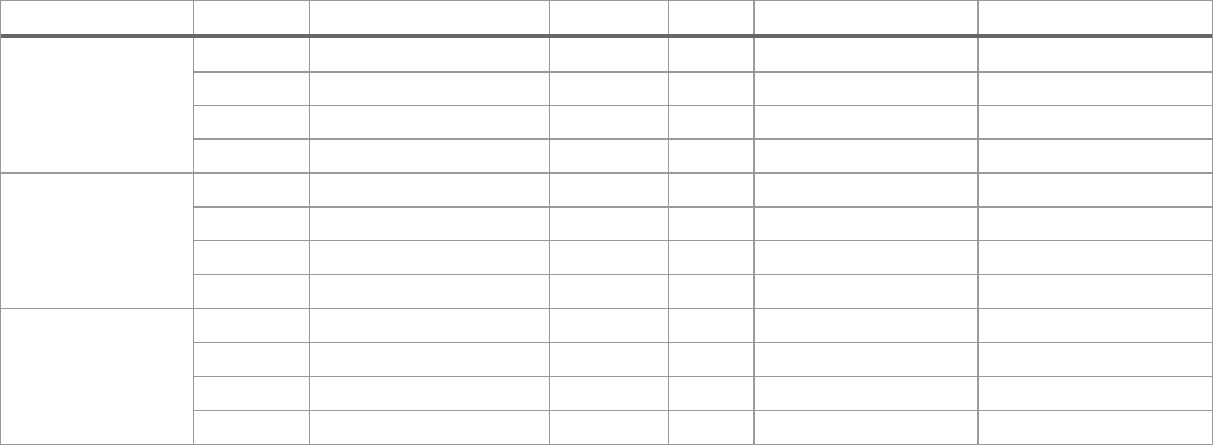


^1^Percentage of the DMC count calculated by (#DMCs)/(#Mapped CpGs) * 100.

^2 ,3^Number of hypo-methylated and hyper-methylated DMCs receptively.

**Table S12.** List of genes that have multiple DMCs in the promoter (P) regions.

| **Comparison** | **Gene ID^1^** | **Gene symbol^2^** | **Gene name** | **#DMCs^3^** |  |
| --- | --- | --- | --- | --- | --- |
| **1C+ vs Ctrl** | 100195955 | rn182 | RING finger protein 182 | 6 (0/6) |  |
|  | 106572013 | LOC106572013 | uncharacterized LOC106572013 | 4 (0/4) |  |
|  | 106602923 | LOC106602923 | serine/threonine/tyrosine-interacting protein | 3 (0/3) |  |
| **1C++ vs Ctrl** |  |  | A-like |  |  |
|  | 100195955 | rn182 | RING finger protein 182 | 4 (0/4) |  |
|  | 100196228 | yars | tyrosyl-tRNA synthetase | 4 (4/0) |  |
| **1C++ vs 1C+** | 106605303 | LOC106605303 | lysyl oxidase homolog 3-like | 3 (0/3) |  |
|  | 100195786 | nca11 | Neural cell adhesion molecule 1-A | 3 (3/0) |  |
|  | 106572013 | LOC106572013 | uncharacterized LOC106572013 | 4 (4/0) |  |
|  | 106588671 | LOC106588671 | DNA topoisomerase 2-beta-like | 2 (0/2) |  |


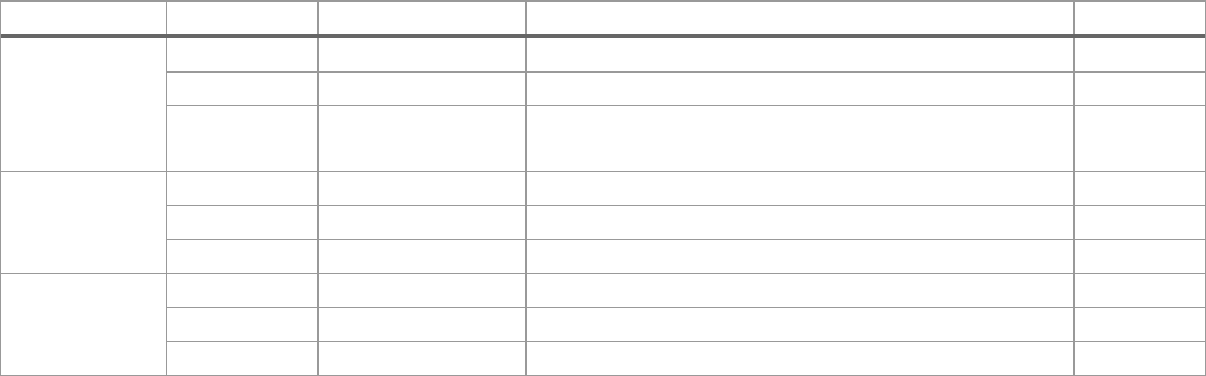


^1,2^Gene ID and gene symbol from NCBI,

^3^Number of DMCs identified in the promoter region with (hypo-methylated/hyper-methylated).

**Table S13.** List of genes that have multiple DMCs in the RS (5K) regions.

| **Select^1^** | **Comparison** | **Gene ID^2^** | **Gene symbol^3^** | **Gene name** | **#DMCs^4^** |  |
| --- | --- | --- | --- | --- | --- | --- |
| **Direct** | 1C+ vs Ctrl | 106574560 | LOC106574560 | cysteine/serine-rich nuclear | 5 (5/0) |  |
|  |  |  |  | protein 3-like |  |  |
|  |  | 106577634 | LOC106577634 | uncharacterized LOC106577634 | 6 (0/6) |  |
|  |  | 106586627 | LOC106586627 | vang-like protein 1 | 5 (0/5) |  |
|  | 1C++ vs Ctrl | 106577634 | LOC106577634 | uncharacterized LOC106577634 | 4 (0/4) |  |
|  |  | 106604632 | LOC106604632 | WD40 repeat-containing protein | 3 (0/3) |  |
|  |  |  |  | SMU1 |  |  |
|  |  | 106610962 | LOC106610962 | transcription factor IIIB 90 kDa | 4 (4/0) |  |
|  |  |  |  | subunit-like |  |  |
|  | 1C++ vs 1C+ | 106586604 | tbx15 | T-box 15 | 3 (1/2) |  |
|  |  | 106591533 | btg4 | B-cell translocation gene 4 | 8 (0/8) |  |
| **In-direct** |  | 106609646 | LOC106609646 | hepatocyte growth factor-like | 4 (0/4) |  |
|  | 1C+ vs Ctrl | 106591533 | btg4 | B-cell translocation gene 4 | 2 (2/0) |  |
|  |  | 106604632 | LOC106604632 | WD40 repeat-containing protein | 1 (0/1) |  |
|  |  |  |  | SMU1 |  |  |
|  |  | 106610962 | LOC106610962 | transcription factor IIIB 90 kDa | 2 (2/0) |  |
|  |  |  |  | subunit-like |  |  |
|  | 1C++ vs Ctrl | 106609646 | LOC106609646 | hepatocyte growth factor-like | 1 (0/1) |  |
|  | 1C++ vs 1C+ | 106574560 | LOC106574560 | cysteine/serine-rich nuclear | 2 (0/2) |  |
|  |  |  |  | protein 3-like |  |  |
|  |  | 106604632 | LOC106604632 | WD40 repeat-containing protein | 2 (0/2) |  |
|  |  |  |  | SMU1 |  |  |


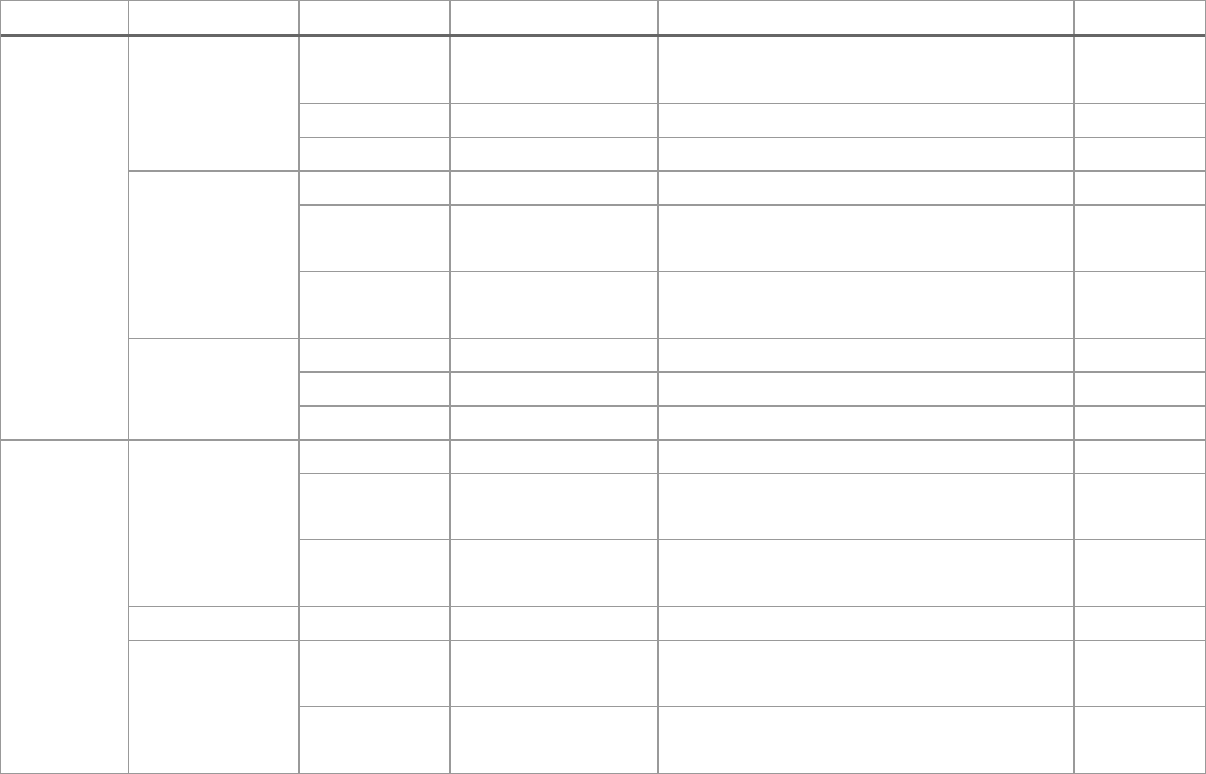


^1^”Direct” and “In-direct” show how the genes are identified. Direct selection is liked to top 3 genes when the genes are sorted by the number of DMCs by descendent order within one of the three comparisons. In-direct selection is simply added when a gene is identified by the “direct” selection and has at least one DMC in the RS (5K) region within other comparisons.

^2,3^Gene ID and gene symbol from NCBI,

^4^Number of DMCs identified in the RS (5K) region with (hypo-methylated/hyper-methylated).

**Table S14.** List of genes that have multiple DMCs in the GB (exon) regions.

| **Select^1^** | **Comparison** | **Gene ID^2^** | **Gene symbol^3^** | **Gene name** | **#DMCs^4^** |  |
| --- | --- | --- | --- | --- | --- | --- |
| **Direct** | 1C+ vs Ctrl | 106571647 | st8sia4 | ST8 alpha-N-acetyl-neuraminide | 5 (5/0) |  |
|  |  |  |  | alpha-2,8-sialyltransferase 4 |  |  |
|  |  | 106574559 | LOC106574559 | xin actin-binding repeat-containing | 5 (5/0) |  |
|  |  |  |  | protein 2-like |  |  |
|  |  | 106577636 | LOC106577636 | RING finger protein 186-like | 6 (0/6) |  |
|  | 1C++ vs Ctrl | 106577636 | LOC106577636 | RING finger protein 186-like | 4 (0/4) |  |
|  |  | 106590562 | LOC106590562 | complement C3-like | 4 (4/0) |  |
|  |  | 106608642 | LOC106608642 | CD276 antigen-like | 5 (5/0) |  |
|  | 1C++ vs 1C+ | 106564966 | LOC106564966 | beta-1,3-galactosyl-O-glycosyl- | 4 (0/4) |  |
|  |  |  |  | glycoprotein beta-1,6-N- |  |  |
|  |  |  |  | acetylglucosaminyltransferase 3- |  |  |
|  |  |  |  | like |  |  |
|  |  | 106571647 | st8sia4 | ST8 alpha-N-acetyl-neuraminide | 6 (0/6) |  |
|  |  |  |  | alpha-2,8-sialyltransferase 4 |  |  |
| **In-direct** |  | 106599566 | LOC106599566 | protein LBH-like | 4 (4/0) |  |
|  | 1C+ vs Ctrl | 106590562 | LOC106590562 | complement C3-like | 4 (4/0) |  |
|  |  | 106599566 | LOC106599566 | protein LBH-like | 4 (0/4) |  |
|  | 1C++ vs Ctrl | 106564966 | LOC106564966 | beta-1,3-galactosyl-O-glycosyl- | 3 (0/3) |  |
|  |  |  |  | glycoprotein beta-1,6-N- |  |  |
|  |  |  |  | acetylglucosaminyltransferase 3- |  |  |
|  |  |  |  | like |  |  |
|  |  | 106599566 | LOC106599566 | protein LBH-like | 2 (2/0) |  |
|  | 1C++ vs 1C+ | 106608642 | LOC106608642 | CD276 antigen-like | 4 (4/0) |  |


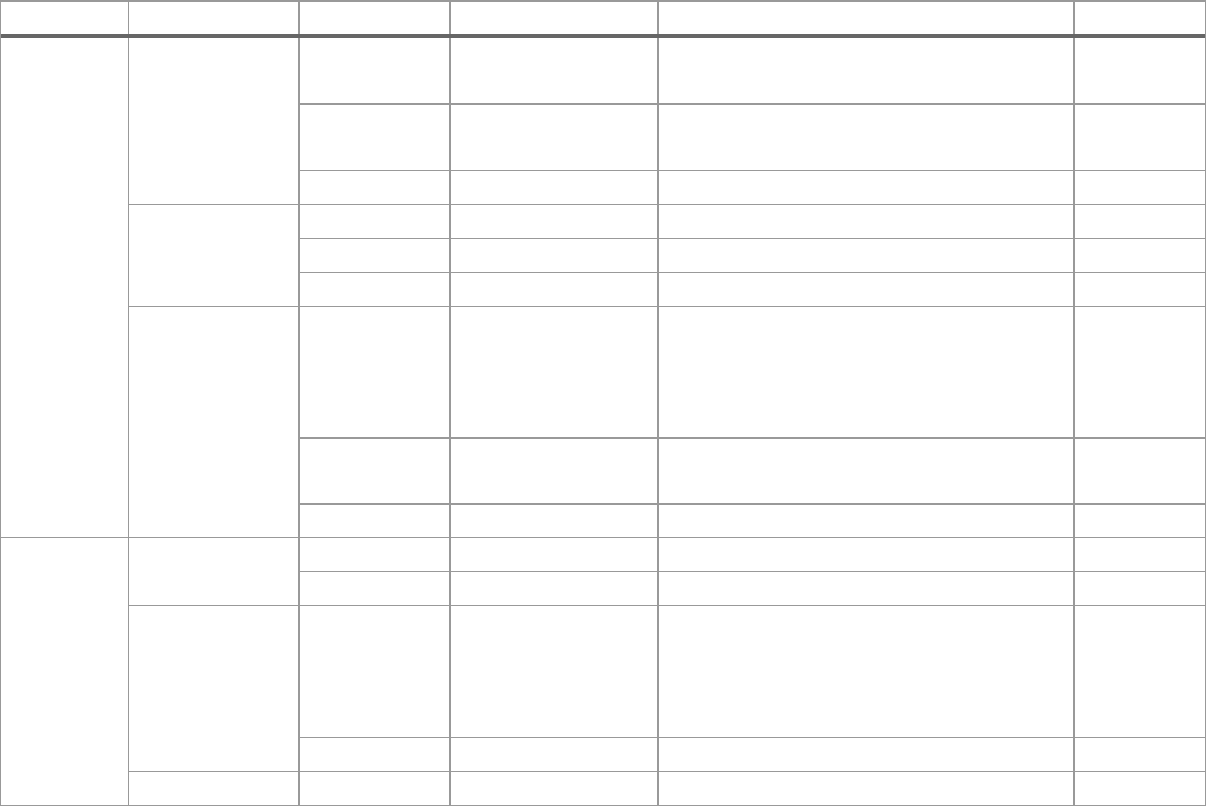


^1^”Direct” and “In-direct” show how the genes are identified. Direct selection is liked to top 3 genes when the genes are sorted by the number of DMCs by descendent order within one of the three comparisons. In-direct selection is simply added when a gene is identified by the “direct” selection and has at least one DMC in the GB (exon) region within other comparisons.

^2,3^Gene ID and gene symbol from NCBI,

^4^Number of DMCs identified in the GB (exon) region with (hypo-methylated/hyper-methylated).

**Table S15.** List of genes that are DEGs and contain DMRs around their TSSs.

| **Comp** | **Region** | **Gene ID^1^** | **Gene symbol^2^** | **Gene name** | **Dist^3^** | **LFC^4^** | **Mdiff^5^** |  |
| --- | --- | --- | --- | --- | --- | --- | --- | --- |
| **1C+ vs** | GB | 106560487 | angptl3 | angiopoietin-like 3 | 553 | -1.53 | 23.80 |  |
| **Ctrl** | (exon) | 106600884 | LOC106600884 | 3-mercaptopyruvate | 971 | 0.34 | 19.57 |  |
|  |  |  |  |  |  |  |  |  |
|  |  |  |  | sulfurtransferase-like |  |  |  |  |
|  | P | 106593742 | LOC106593742 | group 10 secretory | -636 | 1.08 | 15.19 |  |
|  |  |  |  | phospholipase A2-like |  |  |  |  |
|  |  | 106604118 | LOC106604118 | PRELI domain-containing | -829 | 0.31 | 24.21 |  |
|  |  |  |  | protein 1, mitochondrial- |  |  |  |  |
|  |  |  |  | like |  |  |  |  |
|  |  | 106603181 | LOC106603181 | organic solute | -869 | -0.62 | -16.06 |  |
|  |  |  |  | transporter subunit |  |  |  |  |
|  |  |  |  | alpha-like |  |  |  |  |
| **1C++ vs** |  | 106584206 | LOC106584206 | FYN-binding protein 1 | -451 | 1.39 | -16.21 |  |
|  | GB | 106580755 | LOC106580755 | cytochrome P450 2M1- | 280 | -8.41 | 15.14 |  |
| **Ctrl** | (exon) |  |  | like |  |  |  |  |
|  |  | 100380841 | slc3a1 | solute carrier family 3 | 157 | -0.99 | 17.94 |  |
|  |  |  |  | (amino acid transporter |  |  |  |  |
|  |  |  |  | heavy chain), member 1 |  |  |  |  |
|  | P | 106604118 | LOC106604118 | PRELI domain-containing | -829 | 0.34 | 18.16 |  |
|  |  |  |  | protein 1, mitochondrial- |  |  |  |  |
|  |  |  |  | like |  |  |  |  |
|  |  | 106611251 | LOC106611251 | HHIP-like protein 1 | -183 | -0.93 | -16.79 |  |


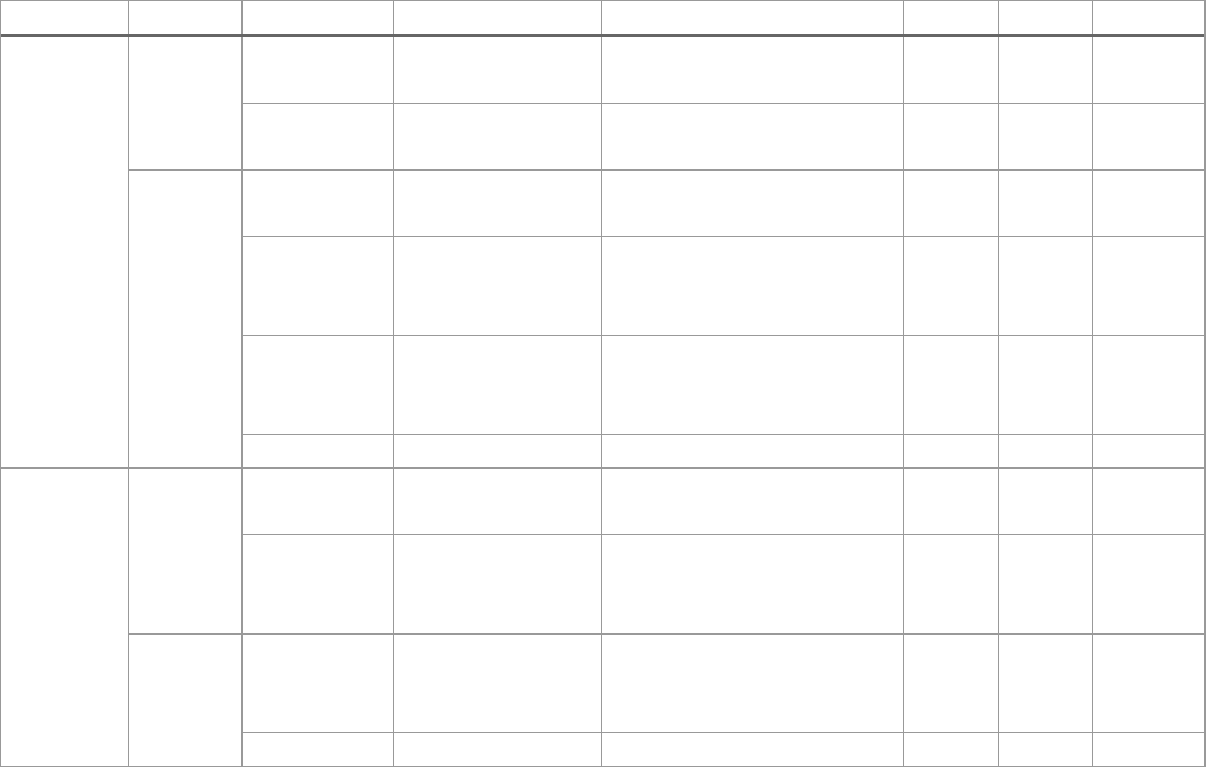


^1,2^Gene ID and gene symbol from NCBI.

^3^Distance from TSS.

^4^Log fold changes of DEGs.

^5^Methylation differences of DMRs.

**Table S16.** List of DEGs from three comparisons: 1C+ vs Ctrl, 1C++ vs Ctrl, and 1C++ vs 1C+.

**File:** 01_degs_total.xlsx (provided in Excel format)

| **Sheets:** | 1C+ vs Ctrl, 1C++ vs Ctrl, 1C++ vs 1C+ | |
| --- | --- | --- |
| **Fields:** | gene_id | Entrez Gene ID from NCBI |
|  | lfc | Log fold change produced by DESeq2 |
|  | padj | Adjusted p-value produced by DESeq2 |
|  | type | RNA type |
|  | gene_symbol | Gene symbol from NCBI |
|  | gene_name | Gene name from NCBI |
|  | orgdb_gene_symbol | Gene symbol from BioConductor OrgDB |
|  | orgdb_gene_name | Gene name from BioConductor OrgDB |
|  | orgdb_alias | Gene alias from BioConductor OrgDB |


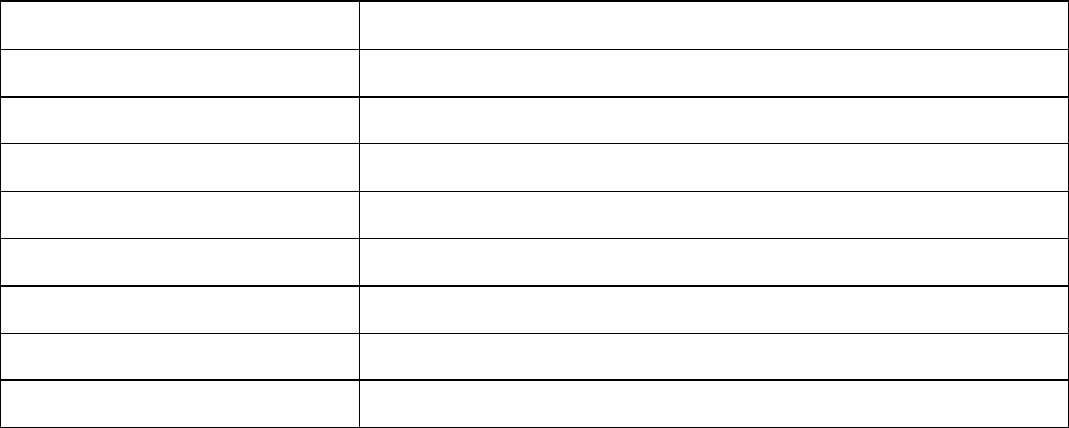


**Table S17.** List of DEGs from three comparisons: 1C+ vs Ctrl, 1C++ vs Ctrl, and 1C++ vs 1C+.

**File:** 02_degs_stringent.xlsx (provided in Excel format)

| **Sheets:** | 1C+ vs Ctrl, 1C++ vs Ctrl |  |
| --- | --- | --- |
| **Fields:** | gene_id | Entrez Gene ID from NCBI |
|  | lfc | Log fold change produced by DESeq2 |
|  | padj | Adjusted p-value produced by DESeq2 |
|  | gene_symbol | Gene symbol from NCBI |
|  | gene_name | Gene name from NCBI |


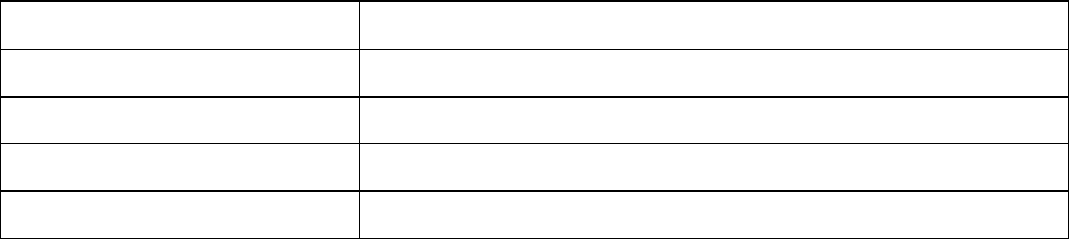


**Table S18.** List of DEGs from three comparisons: 1C+ vs Ctrl, 1C++ vs Ctrl, and 1C++ vs 1C+.

**File:** 03_degs_stringent_relaxed.xlsx (provided in Excel format)

| **Sheets:** | 1C+ vs Ctrl, 1C++ vs Ctrl, 1C++ vs 1C+ | |
| --- | --- | --- |
| **Fields:** | gene_id | Entrez Gene ID from NCBI |
|  | lfc | Log fold change produced by DESeq2 |
|  | padj | Adjusted p-value produced by DESeq2 |
|  | gene_symbol | Gene symbol from NCBI |
|  | gene_name | Gene name from NCBI |


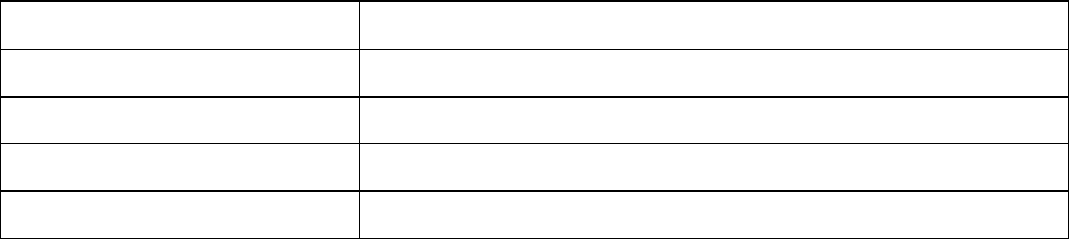


**Table S19.** List of DEG clusters identified by DBSCAN.

**File:** 04_dbscan_clusters.xlsx (provided in Excel format)

**Sheet:** DBSCAN


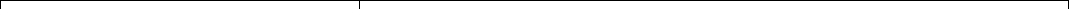


| **Fields:** | gene_id | Entrez Gene ID from NCBI |
| --- | --- | --- |
|  | cluster | Cluster name identified by DBSCAN |
|  | type | RNA type |
|  | gene_symbol | Gene symbol from NCBI |
|  | gene_name | Gene name from NCBI |
|  | orgdb_gene_symbol | Gene symbol from BioConductor OrgDB |
|  | orgdb_gene_name | Gene name from BioConductor OrgDB |
|  | orgdb_alias | Gene alias from BioConductor OrgDB |


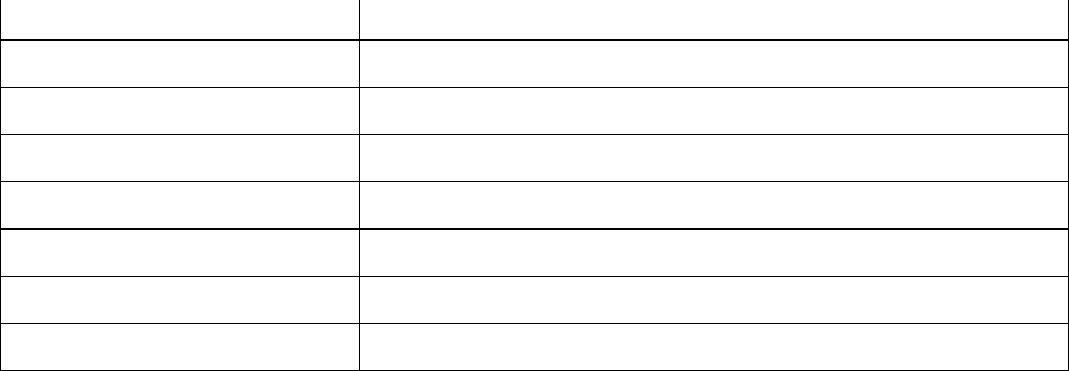


**Table S20.** List of enriched KEGG pathways identified by ORA and GSEA.

| **File:** | 05_kegg_rna.xlsx | (provided in Excel format) |
| --- | --- | --- |
| **Sheets:** | ORA DEG C1, ORA DEG C2 |  |
| **Fields:** | ID | KEGG ID |
|  | Description | KEGG pathway |
|  | GeneRatio | Gene ratio used in ORA calculation |
|  | BgRatio | Back ground ratio used in ORA calculation |
|  | pvalue | p-value calculated by clusterProfiler |
|  | p.adjust | Adjusted p-value calculated by clusterProfiler |
|  | qvalue | Q-value calculated by clusterProfiler |
|  | geneID | Gene IDs of DEGs involvoed in the corresponding KEGG |
|  |  | pathway |
|  | Count | Count of DEGs involvoed in the corresponding KEGG pathway |
| **Sheets:** | GSEA 1C+, GSEA 1C++, GSEA 1C++ vs 1C+ | |
| **Fields:** | Comparison | Comparison result used for GSEA analysis |
|  | ID | KEGG ID |
|  | Description | KEGG pathway |
|  | setSize | The size of gene set used in GSEA calculation |
|  | enrichmentScore | Enrichment score calcualted by clusterProfiler |
|  | NES | Normalised enrichment score calcualted by clusterProfiler |
|  | pvalue | p-value calculated by clusterProfiler |
|  | p.adjust | Adjusted p-value calculated by clusterProfiler |
|  | qvalue | Q-value calculated by clusterProfiler |
|  | rank | Rank calculated by clusterProfiler |
|  | leading_edge | Leading edge analysis performed by clusterProfiler |
|  | core_enrichment | Gene names that contibuted to enrichemnt |


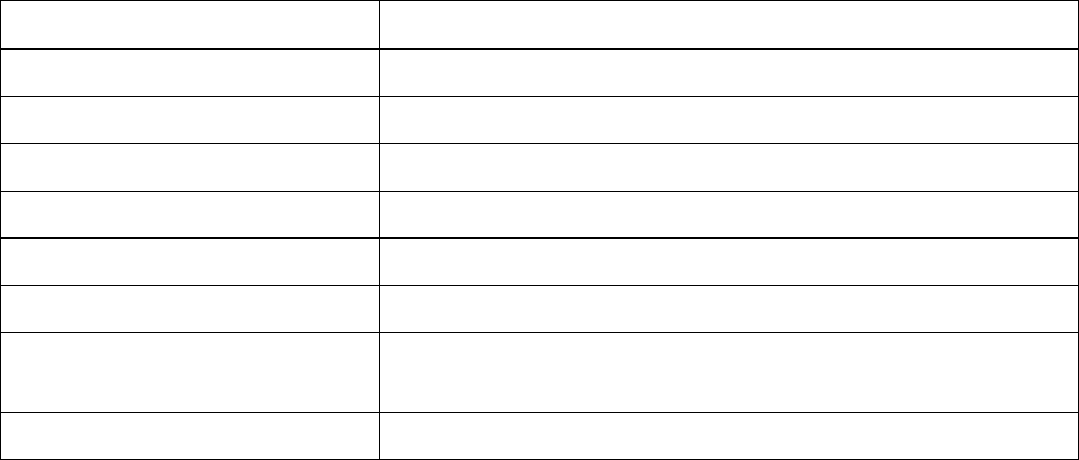

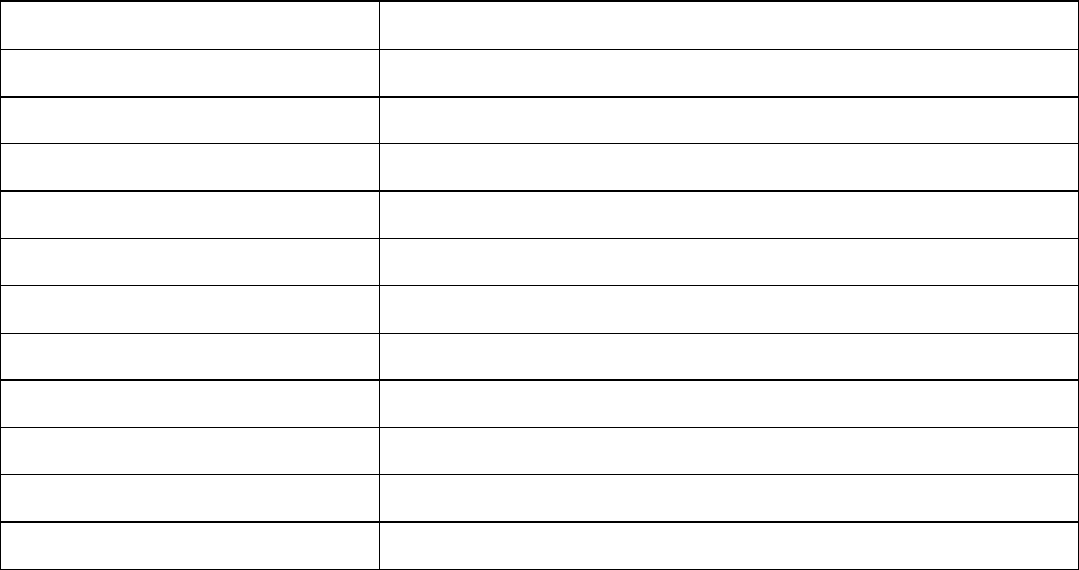


**Table S21.** List of DMCs from three comparisons: 1C+ vs Ctrl, 1C++ vs Ctrl, and 1C++ vs 1C+.

**File:** 06_dmc_md15.xlsx (provided in Excel format)

| **Sheets:** | 1C+ vs Ctrl, 1C++ vs Ctrl, 1C++ vs 1C+ | |
| --- | --- | --- |
| **Fields:** | chrom | Chromosome of DMC |
|  | start | Start position of DMC |
|  | end | end position of DMC |
|  | strand | Strand of DMC |
|  | pvalue | p-value calculated by methylKit |
|  | qvalue | Q-value calculated by methylKit |
|  | meth.diff | Difference of methylation rate (%) |
|  | region | Region |
|  | gene_id | Gene ID from NCBI |
|  | gene_symbol | Gene symbol from NCBI |
|  | gene_name | Gene name from NCBI |
|  | refseq | Refseq ID |
|  | dist | Distance from TSS |


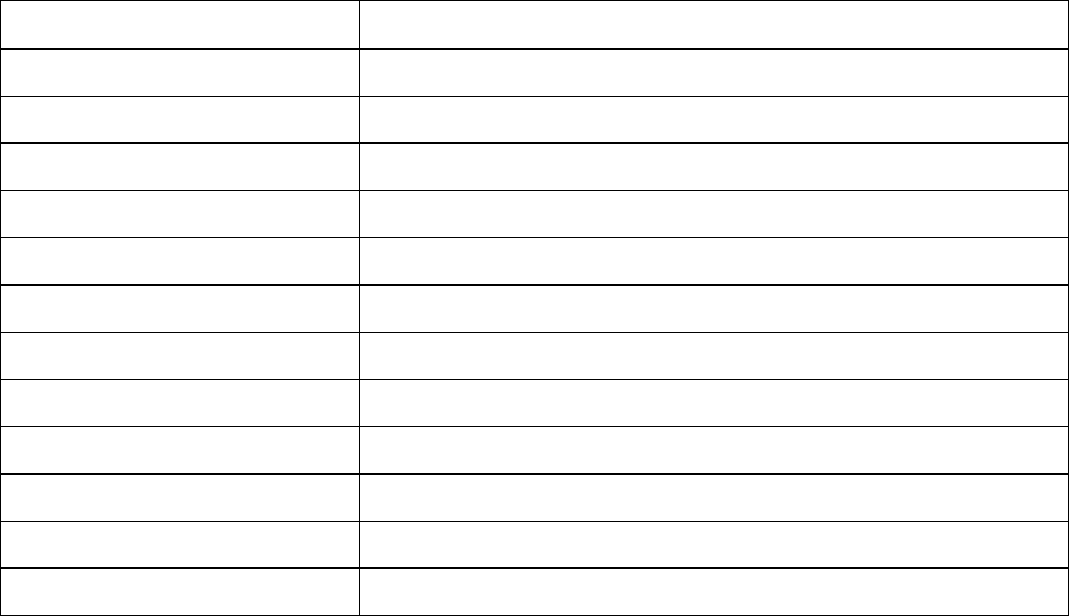


**Table S22.** List of DMRs from three comparisons: 1C+ vs Ctrl, 1C++ vs Ctrl, and 1C++ vs 1C+.

**File:** 07_dmr_md15.xlsx (provided in Excel format)

| **Sheets:** | 1C+ vs Ctrl, 1C++ vs Ctrl, 1C++ vs 1C+ | |
| --- | --- | --- |
| **Fields:** | chrom | Chromosome of DMR |
|  | start | Start position of DMR |
|  | end | end position of DMR |
|  | strand | Strand of DMR |
|  | pvalue | p-value calculated by methylKit |
|  | qvalue | Q-value calculated by methylKit |
|  | meth.diff | Difference of methylation rate (%) |
|  | region | Region |
|  | gene_id | Gene ID from NCBI |
|  | gene_symbol | Gene symbol from NCBI |
|  | gene_name | Gene name from NCBI |
|  | refseq | Refseq ID |
|  | dist | Distance from TSS |


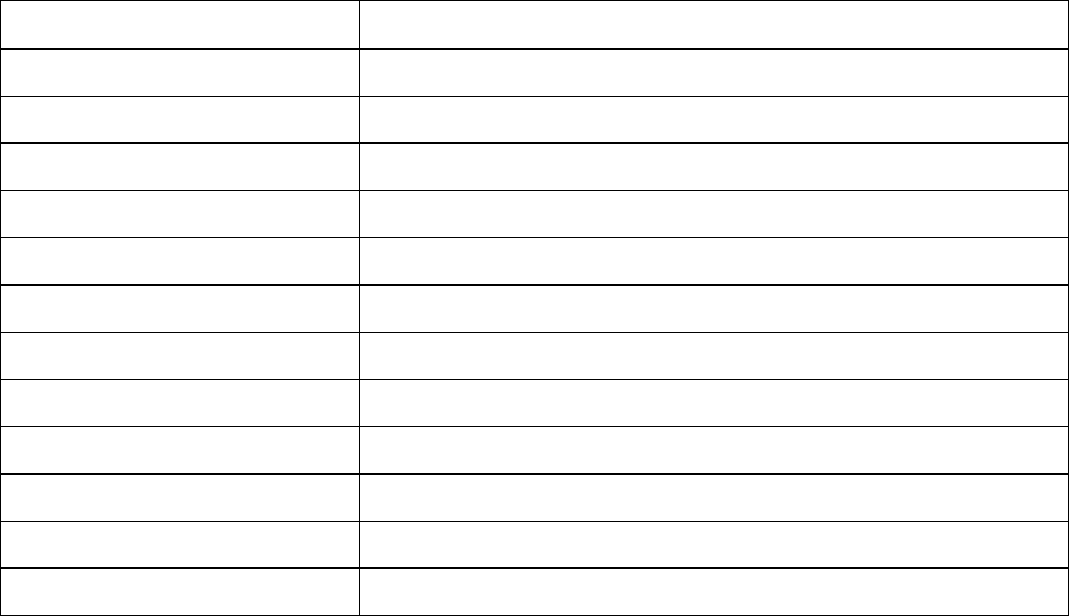


**Supplementary figure**


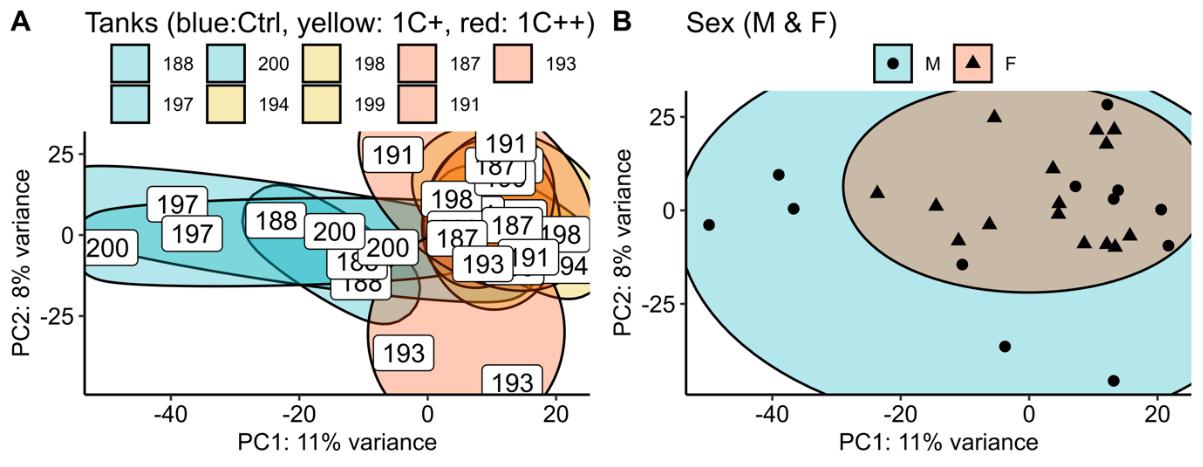


**Fig S1.** Clustering analysis of gene expression differences among tanks and sex.

1. A PCA plot displaying the clusters of nine tanks: Ctrl (blue, semi-transparent), 1C+ (yellow, semi-transparent), and 1C++ (red, semi-transparent) - using 27 RNA-seq samples. The labels represent tank numbers, 188, 197, 200 for the control group, 194, 198, 199 for the 1C+ group, and 187, 191, 193 for the 1C++ group. Top 1000 high variance genes were used as input data. **(B)** A PCA plot displaying the clusters of sex of the fish: M (mele, blue, semi-transparent) and F (female, yellow, semi-transparent. Top 1000 high variance genes were used as input data.


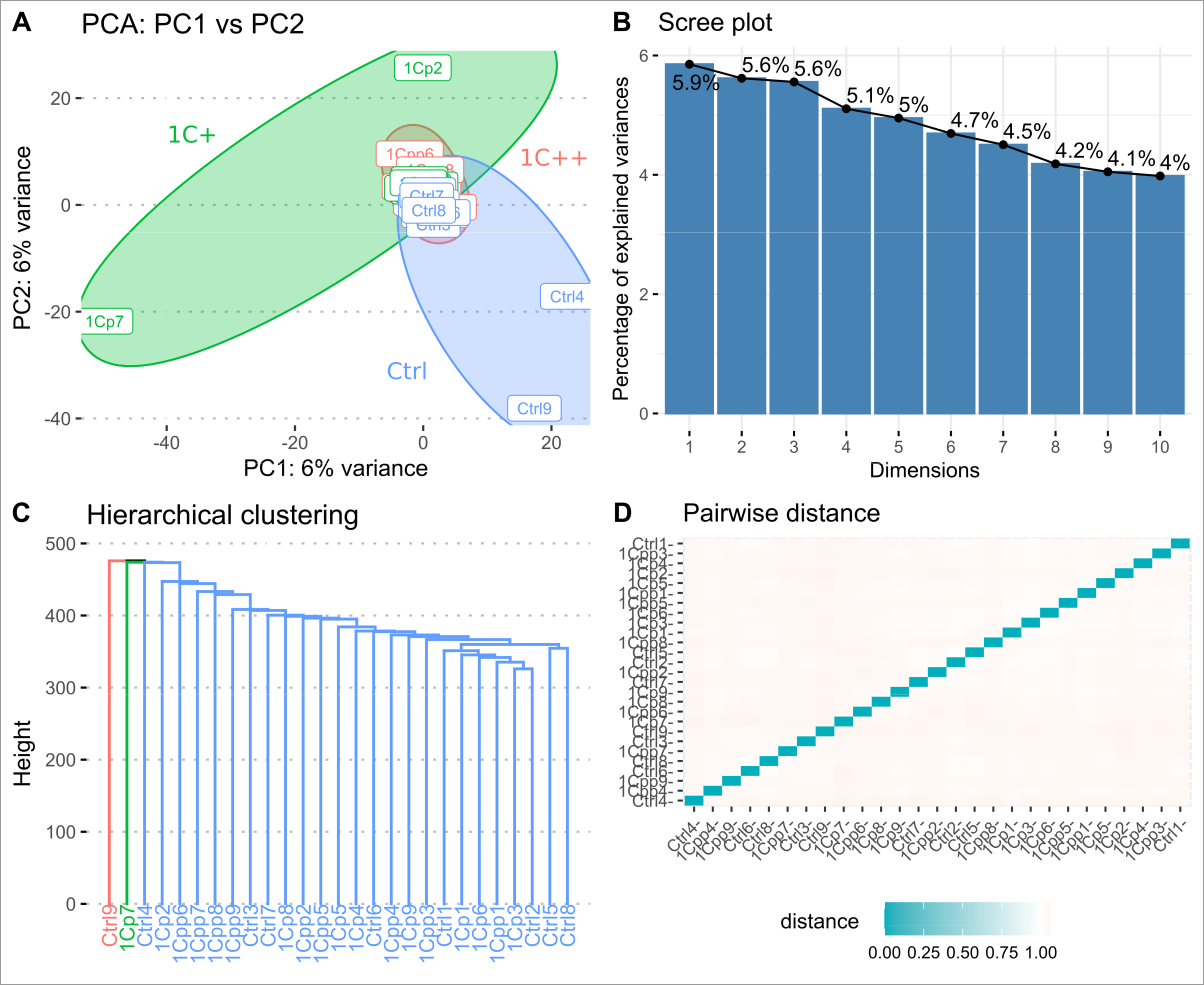


**Fig S2.** Clustering analysis on the methylation rates of mapped CpG.

Four different plots show the results of clustering analysis performed on the CpG sites with top 50% high variances. **(A)** PCA (principal component analysis) plot showing PC1 and PC2 components with three ellipses representing 1C+ (green), 1C++ (red), and Ctrl (blue). **(B)** Scree plot showing the percentage of explained variances for 10 principal components (PC1 ~ PC10). **(C)** The dendrogram showing the result of hierarchical clustering analysis. **(D)** Heatmap showing pairwise distances as the results of pairwise correlation analysis.
